# Delayed Hypersensitivity Reaction: An Increasingly Recognized Complication of Metal-on-Metal Total Disc Replacement

**DOI:** 10.1155/2015/416548

**Published:** 2015-08-09

**Authors:** Mélodie-Anne Karnoub, Fahed Zairi, Rabih Aboukais, Richard Assaker

**Affiliations:** Department of Neurosurgery, Lille University Hospital, CHRU de Lille, 59000 Lille, France

## Abstract

We report the case of a 32-year-old woman who presented with pain recurrence 20 months after she underwent a C5C6 metal-on-metal total replacement. Plan radiographs demonstrated a modification of the shape of the vertebral bodies making the prosthesis more protruding. Then, infection has been ruled out and patch testing revealed a strongly positive reaction for chromium and cobalt. The prosthesis has been removed and a fusion achieved using a cage filled with bone graft. She has been immediately and fully relieved from her pain. We report the radiological signs that enabled early diagnosis and treatment allowing favorable outcome.

## 1. Background

Fusion is the most common treatment of symptomatic cervical disc herniation nonresponsive to medical care. Yet, total disc replacement (TDR) appears to be increasingly used, especially in young population, to avoid degeneration of adjacent discs. Metal-on-metal prostheses have been widely used because they are considered safer and more durable [1]. Most of these implants are composed of an alloy of metal, including cobalt and chromium [2]. Repetitive movements and progressive wear of surfaces cause the release of metal ions, leading to delayed hypersensitivity reaction [3]. Such reaction can induce various symptoms such axial pain, radiculopathy, spinal cord compression, or pseudotumor mass [4]. The diagnosis is even more difficult to establish due to the radiological artefacts caused by the prosthesis. That is, for these reasons, the diagnosis is often established late, which may be responsible for neurological sequelae. We report the clinical and radiological data of a patient for whom the diagnosis was established precociously, enabling early management and favorable outcome.

## 2. Case Report

### 2.1. Medical History

**Figure 1:**
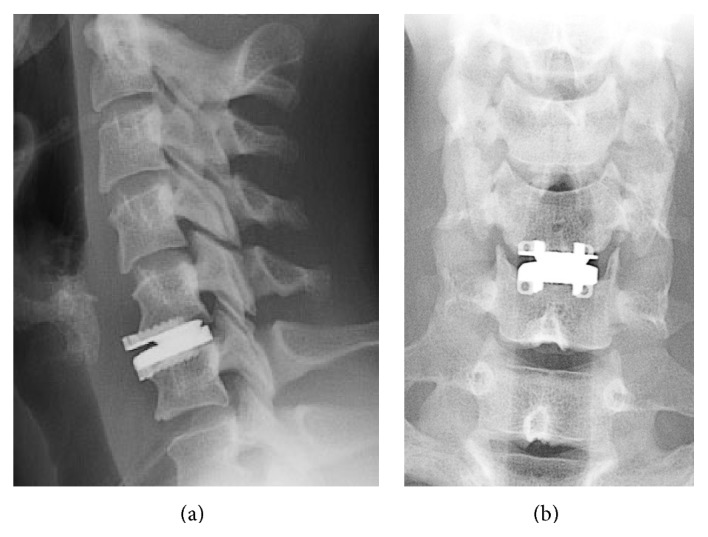
Postoperative lateral (a) and AP (b) radiographs demonstrating that the implant is well positioned on the AP (anteroposterior) view and slightly prominent on the lateral view.

A 32-year-old woman without medical history, suffering from a C6 radiculopathy due to a left sided C5-C6 disc herniation, underwent a metal-on-metal TDR. Surgery and its course were uneventful and the patient was completely relieved for 20 months. Note that the prosthesis was slightly oversized on the lateral view and well centered on the AP (anteroposterior) view (Figure 1). After this delay, she experienced back cervical pain and C6 radiculopathy of 2-month duration, with a complete failure of medical treatment involving analgesics, steroids, and infiltration.

### 2.2. Radiological Findings

**Figure 2:**
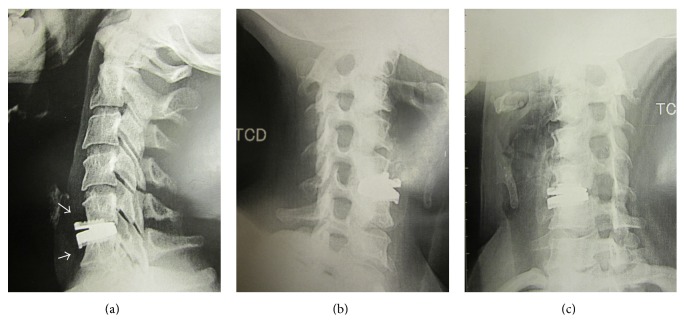
Plan radiographs performed after symptoms recurrence. The endplates adjacent to the prosthesis were shortened, making the prosthesis more protruding (a). The foramina were not narrowed (b and c).

Magnetic resonance imaging did not reveal any other herniation or foraminal stenosis neither at C5-C6 nor at the adjacent levels, yet metal artefacts have distorted the images. Static and dynamic X-rays confirmed the well positioning of the implant and the absence of foraminal stenosis (Figure 2). However, it revealed a modification of the shape of the vertebral bodies. The osteolysis of the anterior wall of the adjacent vertebrae made the endplates shortened and the prosthesis more protruding.

### 2.3. Laboratory Investigations

The complete blood count and the PCR were normal. Then, patch testing was performed that revealed a strongly positive reaction for chromium and cobalt.

### 2.4. Surgery

It was decided to remove the prosthesis and to perform a fusion with a cervical cage filled with bone graft. Particular attention was taken in order not to alter the adjacent endplates. An inflammatory mass with metallic debris, behind the disc and in the foramina, was observed after removal of the prosthesis. A wide decompression of the foramina was performed. The histological analysis revealed a granulomatous reaction with metal wear particles. The culture remained sterile.

As we decided not to use a metal plate to ensure stability, she was fitted with a cervical collar for 3 months. The postoperative course was uneventful and she has been immediately and fully relieved from her pain.

## 3. Discussion

Although fusion remains the gold standard treatment of symptomatic cervical disc herniation, TDR is increasingly used, especially for young patients, in order to keep segmental motion and to reduce the risk of adjacent disc degeneration. Metal-on-metal TDR have been extensively used because they are considered more robust and durable [1]. It is well known that metal-on-metal arthroplasty devices produce metal particles worn off the contact surface of the artificial joint. When particles are released, they undergo corrosion to release ionic compounds. Metal ion exposure may result in activation of macrophages and lymphocytes either directly (nickel) or by binding to endogenous proteins to form metal-protein complexes. This triggers a biologic and immune reaction that aims to eliminate these debris, resulting in resorption of living bone tissue and eventual loosening and failure of the prosthesis [3, 5]. This has been well documented for hip and knee arthroplasty, where osteolysis is considered as one of the foremost problems limiting the survival of current arthroplasty procedures [6–8]. Although host factors are presumed to play a role in the process, which involves a variety of cytokines and mediators, it is likely that surgical factors may be considered. Indeed, repetitive movements and excessive mechanical loads can induce and accelerate the formation of debris and the subsequent immune reaction. In our case, we can hypothesize that the slightly prominent positioning of the prosthesis could have modified the center of rotation and promoted the premature wear of the articular surfaces. The proper placement of the prosthesis and the choice of appropriate materials should be carefully considered when a total disc replacement is suited. Regarding the spine, there are only few cases reported to date [4, 9, 10]. However, the delayed diagnosis due to the lack of knowledge of this disease and the proximity of visceral and neurological structures may be responsible for dreaded and mainly irreversible sequelae [4, 10]. Early diagnosis is required to ensure a good functional outcome. Most of time, MRI and CT scan provide only limited help because of the importance of metal artefacts. Other diagnostic tools must be considered such as scintigraphy, which can show an intense fixation or computed tomography myelogram that can reveal a foraminal and epidural mass [4, 11]. When performed, the cerebrospinal fluid analysis can demonstrate high protein content and lymphocytic pleocytosis [4]. These exams have not been performed in the present case, as the X-rays revealed an osteolysis, resulting in a modification of the shape of the vertebrae, only 2 months after the onset of symptoms. Then, infection has been ruled out and patch testing confirmed the diagnosis of delayed hypersensitivity reaction. Indeed, in a previously nonsensitized patient, a positive patch testing demonstrates the delayed hypersensitivity, as it shows the presence of an immunological reaction induced by the antigen.

The diagnosis of cell mediated hypersensitivity reaction should be considered in cases of unexplained neurologic symptoms or recurrent pain after a latent period, and the modification of the vertebral body shape should be considered as a new early radiological sign. Spine surgeons should be aware of this potential complication of metal-on-metal TDR, requiring reflecting on the legitimacy of their use.

## References

[1] Golish S. R., Anderson P. A. (2012). Bearing surfaces for total disc arthroplasty: metal-on-metal versus metal-on-polyethylene and other biomaterials. *The Spine Journal*.

[2] Grupp T. M., Meisel H.-J., Cotton J. A. (2010). Alternative bearing materials for intervertebral disc arthroplasty. *Biomaterials*.

[3] Zeh A., Planert M., Siegert G., Lattke P., Held A., Hein W. (2007). Release of cobalt and chromium ions into the serum following implantation of the metal-on-metal maverick-type artificial lumbar disc (Medtronic Sofamor Danek). *Spine*.

[4] Zairi F., Remacle J. M., Allaoui M., Assaker R. (2013). Delayed hypersensitivity reaction caused by metal-on-metal total disc replacement. *Journal of Neurosurgery: Spine*.

[5] Agarwal S. (2004). Osteolysis-basic science, incidence and diagnosis. *Current Orthopaedics*.

[6] Campbell P., Ebramzadeh E., Nelson S., Takamura K., De Smet K., Amstutz H. C. (2010). Histological features of pseudotumor-like tissues from metal-on-metal hips. *Clinical Orthopaedics and Related Research*.

[7] Delaunay C., Petit I., Learmonth I. D., Oger P., Vendittoli P. A. (2010). Metal-on-metal bearings total hip arthroplasty: the cobalt and chromium ions release concern. *Orthopaedics and Traumatology: Surgery and Research*.

[8] Fehring K. A., Fehring T. K. (2015). Modes of failure in metal-on-metal total hip arthroplasty. *Orthopedic Clinics of North America*.

[9] Cavanaugh D. A., Nunley P. D., Kerr E. J., Werner D. J., Jawahar A. (2009). Delayed hyper-reactivity to metal ions after cervical disc arthroplasty: a case report and literature review. *Spine*.

[10] Guyer R. D., Shellock J., MacLennan B. (2011). Early failure of metal-on-metal artificial disc prostheses associated with lymphocytic reaction: diagnosis and treatment experience in four cases. *Spine*.

[11] Punt I. M., Austen S., Cleutjens J. P. M. (2012). Are periprosthetic tissue reactions observed after revision of total disc replacement comparable to the reactions observed after total hip or knee revision surgery?. *Spine*.

